# Evaluation of iron, transferrin and ferritin serum levels in patients with severe sepsis and septic shock

**DOI:** 10.1186/cc11031

**Published:** 2012-03-20

**Authors:** M Missano Florido, M Assunção, B Mazza, M Jackiu, F Freitas, A Bafi, F Machado

**Affiliations:** 1 UNIFESP, São Paulo, Brazil

## Introduction

Iron metabolism is altered in critically ill patients leading to hypoferremia. Several studies related it to inflammatory response [1,2]. The present study aims to evaluate iron, transferrin and ferritin serum levels in patients with severe sepsis and septic shock and its association with severity of organ dysfunction.

## Methods

A prospective observational cohort study, unicentric, in a tertiary teaching hospital. From November 2010 to October 2011 patients over 18 years old with severe sepsis or septic shock with up to 72 hours of organ dysfunction were included. Exclusion criteria were blood transfusion or iron supplementation in the last 90 days, previous inclusion and pregnancy. After obtaining informed consent, blood samples were taken at baseline and on day 7. Demographic and APACHE II and SOFA data were also collected. Patients who were transfused with red blood cells between the two periods were excluded from the day 7 sample. Patients were followed until hospital discharge or death.

## Results

Thirty patients were included, with a mean age of 59.6 ± 19.3, APACHE II score 19.1 ± 7.2, SOFA at baseline 8.5 ± 4.0, and most patients had septic shock (63.3%). Baseline iron and transferrin levels were low in 83.3% (14.0 (5.0 to 25.5)) and in 96.7% (94.1 ± 31.6) of the patients, while ferritin was high in 63.3% (954.0 (508.4 to 5,394.0)). In the 19 patients where a day 7 sample was available, variation between baseline and day 7 was statistically significant for transferrin (97.9 ± 37.5 to 132.7 ± 48.3, *P *= 0.013) and ferritin (478.0 (224.5 to 1,741.0) to 376.0 (187.0 to 886.7), *P *= 0.018), while iron levels showed a trend towards increasing levels at day 7 (17.0 (6.5 to 44.3) to 29.0 (21.0 to 54.0), *P *= 0.061). Baseline SOFA score trends to be lower in hypoferrinemic patients (7.7 ± 3.8 vs. 12.4 ± 1.9, *P *= 0.098). The Spearman test showed a weak correlation only between SOFA and iron levels (*P *= 0.008; *r*^2 ^= 0.48).

## Conclusion

Septic patients have low iron and transferrin levels, associated with high ferritin levels, and those levels improved during the course of disease. Low iron levels might be associated with low SOFA scores.
